# Movement Disorders in Cabo Verde: Epidemiology, Access Barriers, and Public Health Implications in an Aging Island Population

**DOI:** 10.3389/ijph.2026.1608728

**Published:** 2026-03-03

**Authors:** Leida C. Tolentino, Joao Filipe G. Monteiro, Cláudia Brito Pires, Antónia Fortes

**Affiliations:** 1 Department of Psychology, Santa Barbara City College, Santa Barbara, CA, United States; 2 Department of Medicine, Brown University, Providence, RI, United States; 3 Center for Neuroscience and Cell Biology, Centre for Innovative Biomedicine and Biotechnology, Universidade de Coimbra, Coimbra, Portugal; 4 Departamento de Neurologia, Hospital Universitário Agostinho Neto, Praia, Cabo Verde

**Keywords:** Parkinson’s disease, neuroepidemiology, aging population, healthcare access, sub-Saharan Africa

## Abstract

**Objectives:**

Movement disorders (MDs), including Parkinson’s disease (PD) and essential tremor, are growing causes of disability globally. Yet, data on MDs in low- and middle-income countries (LMICs), including Cabo Verde, remain scarce. This study aimed to estimate MD prevalence and incidence, identify at-risk groups, and evaluate access to care.

**Methods:**

A multi-site, country-level cross-sectional study was conducted from July to December 2024, including 110 adults with confirmed MDs. Data were obtained via clinical record reviews, interviews, and a structured questionnaire. Analyses included descriptive statistics, multivariable logistic regression, and thematic analysis of open-text responses.

**Results:**

The age-standardized MD prevalence was 17 per 100,000 population (95% CI: 13–20), with higher rates in men [19 (14–25)] and in those over 60 [117 (89–145)]. PD accounted for 79% of cases. While 78% used pharmacotherapy, 70% faced moderate or severe difficulties accessing medication, and 35% of those who sought therapy reported facing barriers. Fifty-one percent experienced a significant life impact.

**Conclusion:**

Findings reveal significant access challenges and rising MD burden, underscoring the need for early diagnosis, decentralized services, and public health system strengthening in LMICs.

## Introduction

Neurological disorders have emerged as the leading cause of disability worldwide, imposing an increasing burden on individuals, families, and health systems, particularly in low- and middle-income countries (LMICs) [1]. Among these, movement disorders (MDs)—which encompass Parkinson’s disease (PD), essential tremor, Huntington’s chorea, dystonias, and other less prevalent forms such as Progressive Supranuclear Palsy (PSP), Multiple Systems Atrophy (MSA), and Corticobasal Degeneration (CBD)—represent a critical but often overlooked cluster of chronic neurological conditions. These disorders are characterized by progressive motor dysfunction, non-motor symptoms, and substantial impacts on quality of life, independence, and societal participation [2]. Globally, PD is the fastest-growing neurological disorder, with estimates indicating a doubling of cases from 6.9 million in 2015 to 14.2 million by 2040 [3]. This trend is largely driven by demographic transitions, particularly the aging of populations, combined with environmental exposures, lifestyle factors, and improved survival from other causes [4].

In Africa, the MD burden is expected to rise sharply due to demographic shifts, including increasing life expectancy and urbanization [5]. However, data on MD prevalence, incidence, and health system responses remain severely limited across the continent, with most available studies concentrated in a few countries and largely restricted to single-center clinical series [6, 7]. These data gaps hinder the development of evidence-informed public health policies, the planning of neurological services, and the allocation of resources needed to meet the growing demand for diagnosis, treatment, and care. This reflects broader challenges faced by LMIC health systems, where 82% of global deaths attributable to neurological conditions occur, and where neurological disorders—traditionally seen as secondary to communicable diseases—are increasingly emerging as a priority area for action, yet remain under-recognized, underfunded, and poorly integrated into national health plans and non-communicable disease (NCD) strategies [1].

Cabo Verde, a small archipelago LMIC off the West African coast, divided into the *Barlavento* and *Sotavento* regions, exemplifies these challenges. With a rapidly aging population (current life expectancy of 80.5 years for women and 73 years for men) and epidemiological transitions underway, MDs are likely to pose increasing demands on the country’s health system [8]. Despite having one of the highest physician densities in sub-Saharan Africa [9], specialized neurological services remain highly centralized, concentrated on the island of Santiago, with no neurologists formally specialized in MDs, and no structured care pathways for these conditions. Furthermore, access to effective pharmacological treatments remains limited due to cost, bureaucratic importation processes, and the unavailability of newer medications within the national formulary, forcing patients to rely on special authorizations for importation or out-of-pocket purchases abroad [10]. Access to essential multidisciplinary therapies such as physiotherapy, speech therapy, and psychological support is also fragmented, underfunded, and geographically inaccessible to many, particularly outside the capital [11].

Prior to this study, no national data existed on the epidemiology of MDs in Cabo Verde. The only available study was a small, single-center retrospective analysis of clinical records from an outpatient neurology service in the capital, reporting on 77 patients, 84% of whom had PD (unpublished data). This limited dataset highlighted the need for more comprehensive epidemiological research to inform national health policy and resource planning. Furthermore, the voices and lived experiences of MD patients and their caregivers, including their perspectives on barriers to care and quality of life, remained undocumented.

The present study aimed to address these critical gaps by conducting the first multi-site, country-level epidemiological survey of MDs in Cabo Verde, encompassing major public hospitals, regional health delegations, and private clinics across several islands. By generating this baseline evidence, the study seeks to support national public health strategies aimed at integrating neurological disorders into broader NCD and aging policies, improving health system responses, and informing advocacy efforts toward more equitable and inclusive care for chronic neurological conditions in LMIC settings. Specifically, the study sought to (a) estimate the age-standardized prevalence and incidence of MDs among adults in Cabo Verde, (b) identify demographic and clinical profiles, and groups at higher risk, and (c) explore the key barriers and systemic challenges related to access to diagnosis, treatment, and supportive care from the perspective of affected individuals and their families.

## Methods

### Study Design and Setting

This study adopted a mixed-methods approach combining quantitative and qualitative data to characterize adults diagnosed with MDs across Cabo Verde, while recognizing the value of integrating patients’ lived experiences with epidemiological data to inform comprehensive, people-centered public health interventions. The design was cross-sectional for prevalence estimation and longitudinal for incidence assessment (time series analysis).

Cabo Verde is an archipelago of ten islands (nine inhabited), located in the Atlantic Ocean off the coast of West Africa. The country has an estimated population of 509,078 individuals, with approximately 77% living in urban areas and a growing aging population currently representing 7.2% of the total population [12]. The health system comprises two central hospitals, four regional hospitals, 18 local health units, and 18 health centers, complemented by several private clinics, mainly located in the capital and other urban areas [13]. Despite this infrastructure, neurological care remains centralized, with only five practicing neurologists in the entire country, all located in the capital city of Praia (Santiago island). There are currently no MD specialists in Cabo Verde.

### Study Population and Recruitment

Eligible participants were adults aged 18 years or older with a confirmed clinical diagnosis of a MD, including PD, essential tremor, Huntington’s chorea, PSP, MSA, CBD, or dystonia. Inclusion criteria required documentation of diagnosis from a licensed neurologist, geriatrician, or general practitioner with neurological training, confirmed through established, validated international diagnostic methods and procedures such as imaging, electrophysiological studies, or neurological examination, and when appropriate biochemical and genetic testing. Exclusion criteria were limited to individuals without a confirmed clinical diagnosis and those under the age of 18.

Participants were identified through hospital records, clinic registries, and health-center databases. Recruitment took place across four hospitals with neurology services—Teaching Hospital Dr. Agostinho Neto and Regional Hospital Santa Rita Viera (Santiago, Sotavento); Central Hospital Dr. Batista de Sousa (São Vicente, Barlavento); and Central Hospital Dr. João Morais (Santo Antão, Barlavento). Additional participants were recruited from four private clinics (MULTIMÉDICA and Praia Clínica on Santiago; Medicentro and URGIMED on São Vicente) and two health centers (Ribeira Grande and Porto Novo on Santo Antão). These sites were selected for their neurology services, documented MD cases, geographic coverage, and patient volume. The two largest referral hospitals—Dr. Agostinho Neto and Dr. Batista de Sousa—were especially important because MD patients are routinely referred there for continued care and diagnosis.

This strategy resulted in data collection from seven out of nine islands, ensuring representation from both island regions (Barlavento and Sotavento), across urban and rural areas, and multiple levels of healthcare. Recruitment and data extraction occurred from 8 July to 28 December 2024, during which all available clinical records, patient-organization lists, and online survey responses were reviewed to identify confirmed MD cases.

Record-review procedures differed by facility: in sites with paper archives, investigators manually reviewed all patient files (A–Z), while in sites with electronic systems, patient lists were generated through neurology departments. This combined approach ensured comprehensive coverage and reduced the likelihood of missing cases.

### Sample Size and Calculation

Because no prior population-level data existed on MDs in Cabo Verde, a formal sample size calculation was not feasible. The study therefore adopted an exhaustive sampling strategy, including all available and confirmed MD cases identified during the study period (8 July–28 December 2024) from public and private healthcare facilities, complemented by patient organization records and self-reported cases through the online survey. This yielded a total analytical sample of 110 adults, across seven out of nine islands, which represents all known diagnosed MD cases in the country at the time of data collection. The approach aligns with recommendations for epidemiological studies in rare or underreported conditions in small-population settings.

### Quantitative Component

#### Data Sources and Collection

Participants were recruited through total population (exhaustive) sampling, aiming to capture all clinically diagnosed MD cases nationwide across both public and private sectors. Participants were identified through three complementary strategies to enhance comprehensiveness and ensure the inclusion of patients from both public and private healthcare settings, reflecting the fragmented care pathways prevalent in Cabo Verde:

Systematic clinical record review: Clinical records were systematically reviewed from four public hospitals, two regional health delegations, and four private clinics with attending neurologists. This approach aimed to capture the maximum number of diagnosed MD cases from both institutional and ambulatory settings. These facilities were selected because they had either neurology services or active inpatient and outpatient registries for neurological disorders. Two of the six public hospitals in the country were not included due to the absence of neurologists and unavailability of complete medical record systems during the study period. Consequently, the analysis covered the principal referral hospitals and regional delegations representing all major island groups (Barlavento and Sotavento), but not every hospital facility nationwide.

Structured interviews: These were conducted either in person or by telephone with individuals identified through clinical records or referred by neurologists, primary care physicians, or the national patient support organization Fundação Doenças do Movimento em Cabo Verde (Fundação DDM-CV). Interviews were conducted by trained researchers and clinicians; no audio was recorded.

Online survey: An anonymous online survey was disseminated via the DDM-CV Foundation website, email outreach, and social media platforms.

All data sources were cross-referenced and harmonized to avoid duplication, using four key identifiers (identification number, date of birth, sex, and municipality of residence).

#### Data Collection Tools and Procedures

Data were collected using three complementary tools:

1. Clinical record abstraction form: Developed to extract standardized data on diagnosis, date of diagnosis, comorbidities, medication use, imaging studies, and healthcare utilization.
2. Structured questionnaire: A 48-item questionnaire was developed by the multidisciplinary research team, drawing from validated international instruments including the WHO Disability Assessment Schedule (WHODAS 2.0), the International Classification of Functioning, Disability and Health (ICF), and public health instruments from the US Department of Health and Human Services and NIH’s TAPS tool [14–16] (Supplementary Material). The questionnaire included dichotomous and Likert-scale items and one open-ended question, and assessed six domains: sociodemographic characteristics, clinical diagnosis and treatment history, access to medications and therapies, daily functioning and quality of life, social and emotional wellbeing, and exposure to risk factors (e.g., occupational exposures, smoking, alcohol use). The questionnaire was pilot-tested with three individuals (two from the DDM-CV Foundation and one clinical psychologist) to evaluate flow, clarity, cultural appropriateness, and technical issues. These participants were excluded from the main sample because they only provided feedback and were not MDs patients.
3. Open-text responses: The survey included one open-ended question inviting participants to describe their experiences living with a MD and the main challenges they encountered in care and daily life.

The questionnaire was administered online using the secure platform Sogolytics© [17]. Two questionnaire versions were developed: a public version without identifiers and a researcher-only version used for structured interviews and clinical record reviews. Paper forms were available for sites with limited connectivity and later digitized. Structured interviews lasted 30–45 min and were conducted in person at clinical sites or by phone. Clinical record reviews, conducted on-site by trained researchers, took 15–25 min depending on record completeness and whether files were paper-based or electronic.

#### Outcome Measures and Operational Definitions

Both crude and age-standardized incidence rates were calculated annually (2022–2024) to evaluate temporal trends and isolate the effects of demographic aging on disease dynamics.

Prevalence: Calculated as the number of existing MD cases per 100,000 population, age-standardized using the 2021 national census as the standard population [8].

Incidence: Defined as the number of new MD diagnoses per 100,000 inhabitants in a given year, also age-standardized. Prevalence and incidence estimates were based on the review of existing clinical records rather than population screening and should be interpreted accordingly. Time-series incidence rates were calculated using available population denominators from national censuses (1986–2021) provided by the Instituto Nacional de Estatística, enabling the assessment of long-term trends standardized by age and sex. To define the incident cohort, each participant’s clinical record was reviewed for the documented year of first diagnosis of a movement disorder by a licensed neurologist or physician with neurological training. A case was classified as an incident if the diagnosis was first established and clearly recorded in the patient’s medical file during the reference year (e.g., 2022–2024) and no prior diagnosis of the same condition was identified in earlier records. Records were examined for previous consultations, follow-up visits, or medication entries to ensure that no earlier diagnosis existed. Cases identified from multiple sources (e.g., public hospitals, regional delegations, or private clinics) were cross-verified using four unique identifiers (national ID, birth date, sex, and municipality) to avoid duplication and misclassification. Only the first documented diagnosis date was used for incidence estimation. The definition aligns with standard epidemiological criteria distinguishing newly diagnosed (incident) from existing (prevalent) cases in registry-based studies.

#### At-Risk Population (Denominator)

The at-risk population used to calculate both prevalence and incidence rates corresponded to the adult population of Cabo Verde as enumerated in the 2021 national census conducted by the Instituto Nacional de Estatística. Age- and sex-specific denominators were derived from the census distribution for each stratum, and national rates were age-standardized using this same reference population in accordance with the WHO standard method for age standardization [18]. Although the study drew cases from a network of hospitals and clinics rather than population screening, it included all major public and private neurological centers across both regions (Barlavento and Sotavento), thereby maximizing national coverage. Because all neurologists practicing in Cabo Verde are located within these institutions, the sample is considered broadly representative of the national distribution of diagnosed movement disorder cases. However, as coverage of rural and remote islands remains incomplete, the resulting prevalence and incidence estimates may underestimate the true burden. To evaluate representativeness, we compared the demographic profile of the study participants with national census data and found similar distributions by sex, mean age, and urban–rural residence.

#### Temporal Dynamics and Age Effects

Age-standardized incidence rates remained stable across the three most recent years, at 5, 4, and 4 cases per 100,000 inhabitants in 2022, 2023, and 2024, respectively (95% CI for all years: 0–10). However, crude counts of newly diagnosed cases increased modestly during the same period (by 25% in 2022 and 10% in 2023), suggesting an expanding number of diagnosed individuals in an aging population rather than a true rise in disease risk.

Although prevalence was formally estimated for 2024, the consistent number of new diagnoses alongside a visible growth in the number of living cases indicates increasing survival and reduced competing mortality. This pattern—stable standardized incidence but rising crude case counts—is compatible with demographic aging and improvements in chronic disease management in Cabo Verde. The age-stratified analysis confirmed that incidence was highest among individuals aged ≥60 years, supporting the interpretation that population aging is a key driver of prevalence trends.

Access to care: Measured through self-reported difficulty in accessing medications and therapies, categorized as none, mild, moderate, or severe.

Quality of life impact: Assessed using an index composed of 29 indicators across cognitive, physical, emotional, financial, and social domains.

Lived experience: Thematic categories emerging from open-text responses were analyzed using Natural Language Processing (NLP) techniques and grouped into key domains such as emotional distress, barriers to healthcare, and functional limitations.

### Data Management and Statistical Analysis

Data from clinical records, questionnaires, and interviews were entered into a centralized database and cleaned for quality and consistency. Quantitative data were analyzed using both descriptive and inferential statistics in SAS 9.4 [18]. Categorical variables were compared using chi-square or Fisher’s exact tests, and continuous variables using independent-samples *t*-tests or Mann–Whitney *U* tests when normality assumptions were not met**.** Age-standardized prevalence and incidence rates were estimated using the binomial method with 95% confidence intervals assuming normal distribution [19]. Multivariable logistic regression models were used to identify predictors of (a) difficulty accessing medications or therapies and (b) strong or extreme quality-of-life impact. Models were adjusted for sex, age, region, urban/rural residence, income, comorbidities, and exposure to hazardous materials. Regression results were expressed as odds ratios (OR) with 95% confidence intervals (CI), and *p* < 0.05 was considered statistically significant. For time-trend analysis of incidence, generalized linear models were fitted with sex, age, and region as covariates. Time-series analyses of incidence were performed using generalized linear models with a Poisson distribution and log link, modeling the number of new cases per year as a function of calendar year (1986–2024) and adjusting for population size as an offset term. Incidence rate ratios (IRR) with 95% confidence intervals (CI) and corresponding p-values were estimated to assess annual trends overall and by sex. Model fit and overdispersion were evaluated; when overdispersion was present, a quasi-Poisson correction was applied.

### Qualitative Component

Open-text responses were analyzed using NLP methods to identify frequent terms and phrases, followed by manual thematic validation by two independent reviewers. Themes were then categorized and quantified. All statistical analyses were performed using SAS software version 9.4. The study followed the STROBE checklist for cross-sectional studies and the COREQ checklist for qualitative components to ensure transparent and standardized reporting.

Quantitative and qualitative findings were integrated at the interpretation stage to provide a comprehensive, mixed-methods understanding of epidemiological patterns and lived experiences of movement disorders in Cabo Verde.

### Ethical Considerations

The study was approved by the National Ethics Committee for Health Research (CNEPS; Ref. 26/CNEPS/2023) and the National Commission for Data Protection (CNPD; Ref. 145/2022/CNPD). Written informed consent was obtained from all participants or their legal representatives. Data was collected using secure platforms with automatic audit trails. All clinical records and personal identifiers were stored separately from survey data in encrypted files, and data were pseudonymized prior to analysis. No financial compensation was provided to participants. The study adhered to the principles of the Declaration of Helsinki and the WHO’s guidance on research involving vulnerable populations and persons with disabilities.

## Results

### Participant Characteristics

A total of 110 adults with a confirmed diagnosis of a MD were included. Participants were predominantly male (54%) with a mean age of 67.6 years (±14.5 SD) and a mean age at diagnosis of 61.0 years (±16.2 SD). The majority (77%) were diagnosed after the age of 50. Sixty percent lived in urban areas, and 61% resided in the Sotavento region, with Santiago Island representing the highest proportion (59%), Table 1.

**TABLE 1:** Demographic characteristics of participants with movement disorders in Cabo Verde, 2024 (N = 110).

Characteristic	N (%)
Current age (mean ± SD)	67.6 ± 14.5 years
<30 years	2 (2.3%)
31–50 years	9 (10.5%)
51–70 years	40 (46.5%)
>70 years	35 (40.7%)
Age at diagnosis (mean ± SD)	61.0 ± 16.2 years
<30 years	2 (3.2%)
31–50 years	12 (19.4%)
51–70 years	30 (48.4%)
>70 years	18 (29.0%)
Male sex	59 (53.6%)
Marital status	
Married	33 (30.0%)
Single	29 (26.4%)
Not reported	48 (43.6%)
Type of residence	
Own home	82 (74.5%)
Residential facility	1 (1.0%)
Not reported	27 (24.6%)
Residence area	
Urban	66 (60.0%)
Rural	39 (35.5%)
Not reported	5 (4.6%)
Sotavento region of residence	66 (60.6%)
Island of residence	
Santiago	64 (58.7%)
São vicente	24 (22.0%)
Santo antão	15 (13.8%)
Sal	2 (1.8%)
Fogo	2 (1.8%)
Boa vista	1 (0.9%)
São nicolau	1 (0.9%)
Health insurance	
Yes	73 (66.4%)
No	18 (16.4%)
Uncertain	8 (7.3%)
Not reported	11 (10.0%)
INPS	66 (61.1%)
Other insurance	9 (8.3%)
Not reported	33 (30.6%)
Education level	
Primary (4th grade)	27 (24.8%)
Secondary (liceu)	9 (8.3%)
Bachelor’s degree	10 (9.2%)
Postgraduate	8 (7.3%)
Not reported	55 (50.5%)
Main occupation	
Employed	21 (19.1%)
Retired	38 (34.6%)
Unemployed due to illness	7 (6.4%)
Not working	5 (4.6%)
Not reported	39 (35.5%)
Monthly household income	
≤10 000 ECV	8 (7.5%)
11–50 000 ECV	11 (10.3%)
51–100 000 ECV	11 (10.3%)
>101 000 ECV	9 (8.4%)
Not reported	68 (60.6%)
Household size (mean ± SD)	2.3 ± 2.6 persons

SD, standard deviation; INPS, Instituto Nacional de Previdência Social [National Social Security Institute]; ECV, Cabo Verdean Escudo. Percentages may not total 100% due to rounding and missing data. 'Not reported' refers to participants who did not provide information for that variable.

Regarding civil status, 30% were married, 26% were single, and 44% did not report their status. Most lived in private residences (75%) while only 1% lived in institutional care. Educational attainment was low, 25% had only primary-level education, 24% completed secondary or higher education; however, 50% did not report. Employment data showed 35% were retired, 19% employed, and 6% unemployed due to health conditions. Household size averaged 2.3 (±2.6) persons. Income data were limited, with only 39% of participants reporting earnings, Table 1.

Informal support was limited: 43% received assistance from family or caregivers, most commonly spouses (18%) and children (25%), while 57% had no regular support, Table 1.

### Clinical Profile

Parkinson’s disease was the most prevalent diagnosis, accounting for 79% of cases, followed by essential tremor (9%) and Huntington’s chorea (8%). MSA and dystonia were each reported in 1% of participants. Sixteen percent had a family history of MD, and 90% were under the care of a neurologist. Comorbidities were common, with a mean of 1.3 (±1.2) per participant. Hypertension (54%), diabetes (26%), and hyperlipidaemia (12%) were the most frequently reported, along with stroke (9%) and chronic pulmonary or renal conditions (5%–6%). Thirty-five percent had been exposed to hazardous materials, Table 2.

**TABLE 2:** Comorbidities, exposures, and clinical characteristics of participants with movement disorders in Cabo Verde, 2024 (N = 110).

Characteristic	N (%)
Comorbidities and exposures	
Number of comorbidities (mean ± SD)	1.3 ± 1.2
No comorbidities	31 (28.2%)
1–2 comorbidities	61 (55.5%)
>2 comorbidities	18 (16.4%)
Type of comorbidity	
Type 1 diabetes	3 (2.7%)
Type 2 diabetes	23 (20.9%)
Diabetes with complications	3 (2.7%)
Hypertension	59 (53.6%)
Hyperlipidaemia	13 (11.8%)
Obesity	4 (3.6%)
Thyroid disorder	3 (2.7%)
Atrial fibrillation	1 (1.0%)
Stroke	10 (9.1%)
Chronic kidney disease	4 (3.6%)
Congestive heart failure	3 (2.7%)
Metastatic cancer	2 (1.8%)
Chronic obstructive pulmonary disease (COPD)	5 (4.6%)
Other comorbidities	13 (11.8%)
Substance use	
Tobacco	10 (9.1%)
Alcohol	20 (18.2%)
Illicit drugs	3 (2.7%)
Prescription drug misuse	9 (8.2%)
Exposure to hazardous materials	19 (34.5%)
Iron	9 (8.2%)
Copper	7 (6.4%)
Pesticides	2 (1.8%)
Other	5 (4.6%)
Clinical characteristics	
Family history of MD	
Yes	18 (16.4%)
No	32 (30.0%)
Don’t know	15 (13.6%)
Not reported	44 (40.0%)
Followed by a neurologist	93 (90.3%)
Type of personal care support	
Spouse	20 (18.2%)
Children	27 (24.6%)
Siblings	4 (3.6%)
Grandchildren	4 (3.6%)
Mother	7 (6.4%)
Caregiver	3 (2.7%)
No one	63 (57.3%)

SD, standard deviation; COPD, chronic obstructive pulmonary disease; MSA, multiple system atrophy; MD, movement disorder. Percentages may not total 100% due to rounding or missing data.

### Diagnostic Imaging

Fifty-seven percent of participants had undergone brain imaging. Computed tomography (CT) was the most common (44%), followed by magnetic resonance imaging (MRI) at 31%. Access to positron emission tomography (PET) scans was rare (3%) as this is not yet available in the country.

### Access to Treatment and Therapy

Seventy-eight percent were receiving pharmacological treatment for MDs, predominantly antiparkinsonian agents (72%). Anticonvulsants and neuroleptics were prescribed less frequently. Access to medication was a major barrier: all of the participants reported difficulty obtaining medications, with 51% rating it as moderate and 19% as severe. Thirty-eight percent received assistance from the National Social Security Institute (INPS) for medications, while 43% were unsure, Table 3.

**TABLE 3:** Availability and accessibility of treatments and therapies for participants with movement disorders in Cabo Verde, 2024 (N = 110).

Characteristic	N (%)
More than one type of brain imaging performed	63 (57.3%)
Type of brain imaging	
Computed tomography (CT)	48 (43.6%)
Magnetic resonance imaging (MRI)	34 (30.9%)
Positron emission tomography (PET)	3 (2.7%)
Other brain imaging	3 (2.7%)
Type of medication	
Antiparkinsonians	79 (71.8%)
Neuroleptics	4 (3.6%)
Anticonvulsants	7 (6.4%)
No medication	24 (21.8%)
Difficulty accessing medications	
Mild	13 (30.2%)
Moderate	22 (51.2%)
Severe	8 (18.6%)
INPS support for medications	
Yes	41 (38.0%)
No	21 (19.4%)
Unknown	46 (42.6%)
Any therapy received	42 (38.2%)
Type of therapy	
Physical therapy	37 (33.6%)
Speech therapy	9 (8.2%)
Nutritional counselling	2 (1.8%)
No therapy received	68 (61.8%)
Reasons for no therapy	
Not recommended	10 (9.1%)
Financial constraints	5 (4.6%)
Difficulty travelling	1 (1.0%)
Discomfort due to heat or pain	4 (3.6%)
Difficulty accessing therapy	
Mild	6 (35.3%)
Moderate	8 (47.1%)
Severe	3 (17.7%)
INPS support for therapy services	
Yes	27 (24.6%)
No	6 (5.5%)
Unknown	77 (70.0%)
Physical activity	41 (37.3%)

INPS, Instituto Nacional de Previdência Social [National Social Security Institute]; CT, computed tomography; MRI, magnetic resonance imaging; PET, positron emission tomography. Percentages may not total 100% due to rounding or missing data.

Thirty-eight percent of participants engaged in rehabilitative therapies: primarily physiotherapy (34%), speech therapy (8%), and nutritional counselling (2%). However, 62% reported not receiving any therapy, mainly due to a lack of referral, financial barriers, or mobility challenges. Among those seeking therapy (44%, 48/110), 35% (17/48) faced difficulties, with 47% of those describing moderate and 18% severe access barriers, Table 3.

### Bivariate and Multivariate Associations

Bivariate analyses revealed significant differences in access and diagnostic patterns across subgroups. Participants residing outside Santiago reported higher frequency of moderate or severe difficulty obtaining medications (p = 0.031). Access to MRI was over four times more likely among those in the Barlavento region than in Sotavento (OR = 4.6; 95% CI 1.1–18.2; p = 0.028). In multivariable models, low household income (<20,000 ECV) and presence of ≥2 comorbidities were independently associated with increased odds of access barriers (adjusted OR = 2.8; 95% CI 1.3–5.9; p = 0.007). Participants aged >60 years and those reporting emotional distress had significantly higher odds of strong or extreme quality-of-life impact (adjusted OR = 3.4; 95% CI 1.4–8.2; p = 0.007).

### Quality of Life and Functional Challenges

**FIGURE 1:**
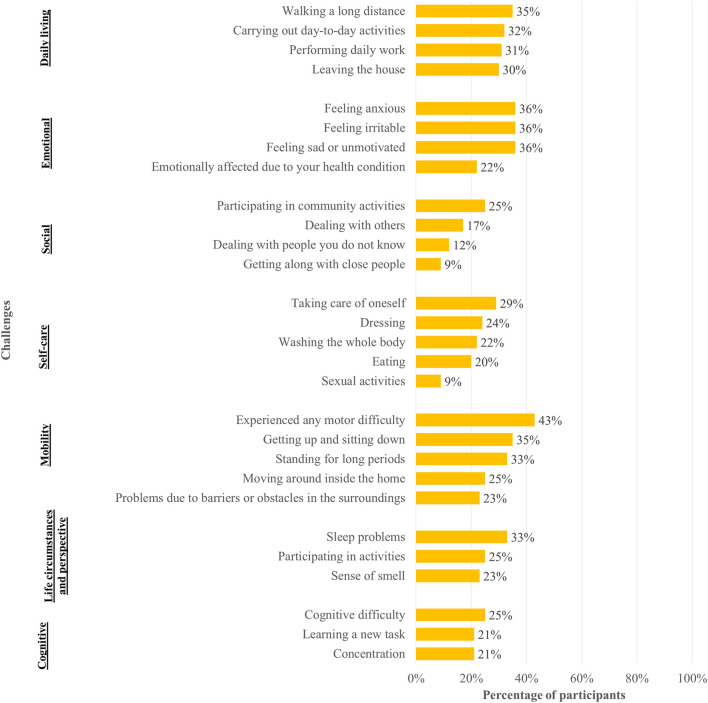
Cognitive, life circumstances and perspective, mobility, and self-care challenges among participants with movement disorders in Cabo Verde (2024). Notes: Data were collected using a structured questionnaire based on validated, standardized scales. Reported challenges among participants with movement disorders in Cabo Verde included emotional difficulties (e.g., sadness, anxiety, irritability), cognitive impairments (e.g., memory and concentration issues), and limitations in social interaction. Participants also described difficulties with physical functioning and daily living activities, including mobility, working, going out, and self-care tasks such as washing, dressing, and eating.

Over half of participants (51%) reported a strong or extreme impact of MDs on their quality of life. Motor impairments were the most frequent functional challenge (43%), followed by sadness or lack of motivation (36%), anxiety (36%), and difficulties with standing and walking (35%). Cognitive issues were also common: 25% reported memory difficulties, and 21% struggled with concentration or learning new tasks, Figure 1.

Self-care activities were affected in a major proportion: 29% had difficulties with personal care, 24% with dressing, and 22% with bathing. Participation in community activities and emotional wellbeing were compromised in 36% of respondents, Figure 1. Financial impact was reported by 22%, with the effects lasting an average of 13.7 days per month.

### Prevalence and Incidence

Prevalence and incidence rates were calculated using the 2021 Cabo Verde national census population [8] as the denominator, with age-standardization applied to allow national comparability.

**FIGURE 2:**
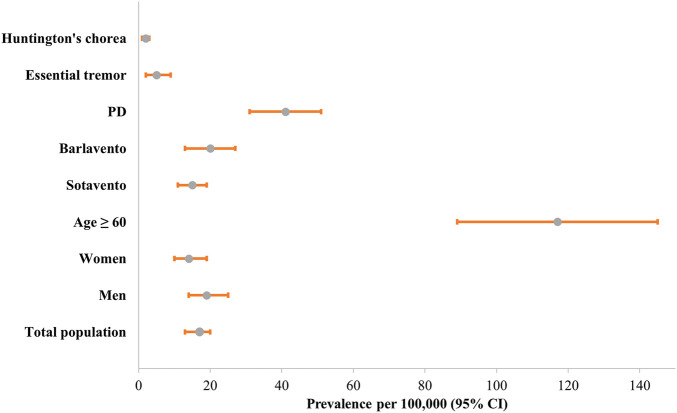
Age-standardized prevalence (per 100,000 population; 95% CI) of movement disorders by demographic and diagnostic group in Cabo Verde (2024). Notes: Prevalence estimates are age-standardized and presented per 100,000 population with corresponding 95% confidence intervals. Data were collected through clinical records and structured questionnaires based on validated scales. Groups include demographic subpopulations (sex, age, and region) and diagnostic categories (Parkinson’s disease, essential tremor, and Huntington’s chorea). Standardization was based on the 2021 Cabo Verde national census population structure.

The national age-standardized prevalence of MDs was estimated at 17 per 100,000 inhabitants (95% CI: 13–20). Prevalence was higher among men (19 per 100,000; 95% CI: 14–25) compared to women (14 per 100,000; 95% CI: 10–19), and markedly elevated in individuals aged 60 years and older, reaching 117 per 100,000 (95% CI: 89–145). Regional differences were evident: prevalence was 15 per 100,000 (95% CI: 11–19) in the Sotavento islands versus 20 per 100,000 (95% CI: 13–27) in Barlavento. Parkinson’s disease had the highest age-standardized prevalence at 41 per 100,000 (95% CI: 31–51), followed by essential tremor (5 per 100,000; 95% CI: 2–9) and Huntington’s chorea (2 per 100,000; 95% CI: 1–3), Figure 2.

**FIGURE 3:**
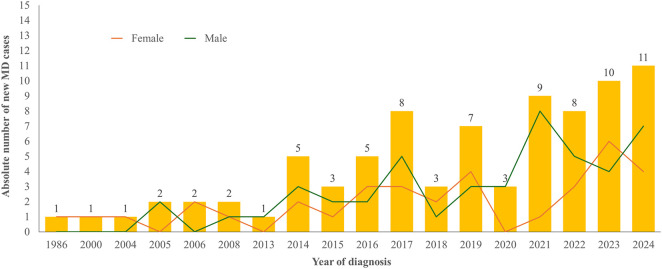
Number of newly diagnosed cases of movement disorders by year and sex, from 1986 to 2024, in Cabo Verde. Notes: Data are derived from clinical records across public and private institutions and reflect national diagnostic patterns. The figure illustrates temporal trends and sex-specific differences in newly diagnosed cases. Despite progressive urbanization and population aging over the same period, the standardized incidence remained largely stable, suggesting that the observed increase in absolute numbers may reflect improved diagnostic capacity and health service access rather than a true rise in disease occurrence.

Incident cases (new diagnoses first recorded between 2022 and 2024) were confirmed through clinical documentation review to ensure that no prior diagnosis existed in earlier years. Age-standardized incidence rates remained stable over the past 3 years: 5 per 100,000 in 2022, and 4 per 100,000 in both 2023 and 2024 (95% CI for all years: 0–10). However, time-series Poisson regression showed a significant upward trend in new diagnoses among men (IRR = 1.17; 95% CI: 1.07–1.30; p = 0.003), indicating an average 17% annual increase, while no significant trend was observed among women (IRR = 1.00; 95% CI: 0.93–1.07; p = 0.97), Figure 3.

### Open-Ended Responses: Lived Experience and Challenges

Of the 110 participants, 68 (62%) provided narrative responses describing their lived experiences with movement disorders. Using natural language processing (NLP), several thematic categories emerged. Emotional distress—characterized by sadness, anxiety, and hopelessness—was the most frequent theme, with many participants linking their emotional state to physical decline and social isolation. Functional limitations in daily activities, such as walking, self-care, and employment, were widely reported, often compounded by loss of independence and fear of falling. Access to medication was a recurring concern, with many citing financial barriers, inconsistent availability, or reliance on international procurement. Respondents also highlighted gaps in specialized care, lack of transport for therapy, and insufficient INPS coverage. A subset of participants expressed feelings of marginalization, describing being stigmatized or pitied. Suggestions included increasing local access to neurologists, improving therapeutic services, expanding financial support, and enhancing public awareness around the burden of movement disorders in Cabo Verde.

Qualitative analysis of open-ended responses revealed six core themes describing the lived experiences of participants: (1) emotional distress and coping; (2) daily functional limitations; (3) barriers to medication access; (4) challenges in accessing healthcare; (5) financial strain; and (6) loss of autonomy. Respondents described how sadness, worry, and anxiety directly affected their health and daily functioning (“It affected me a lot—always worried, feeling down and anxious”). Functional limitations were a recurrent concern (“Tremors make it hard to move; I’m afraid of falling”), as were difficulties accessing medication (“The biggest difficulty in Cabo Verde is the medicine—it’s always missing”). Participants emphasized financial burdens (“We need help paying for medicine”), and dependence on others for basic activities (“I try every day not to depend on others; accepting Parkinson’s was very hard”). Faith and family support were often cited as coping mechanisms (“I speak with God to help me keep going”). These narratives highlight the multifaceted burden of MDs, spanning emotional, physical, financial, and social dimensions.

## Discussion

This first country-level epidemiological study of MDs in Cabo Verde provides critical baseline evidence on the prevalence, incidence, and characteristics of this group of chronic neurological conditions in a small island LMIC setting. The results revealed an age-standardized MD prevalence of 17 per 100,000 inhabitants and an incidence of 4 per 100,000, both of which are substantially lower than global and regional averages [2]. The data also highlighted the disproportionate burden of MDs among older adults and men, as well as the profound barriers to care and therapy access that patients face within the Cabo Verdean health system.

These findings carry significant public health implications, underscoring the growing burden of MDs in the context of demographic aging and fragile health system capacities in LMICs. The study also reveals systemic barriers and gaps in care, which align with broader regional challenges in sub-Saharan Africa, where neurological conditions remain largely under-recognized within national health agendas, despite their increasing contribution to disability and health system costs [7].

Inferential analyses confirmed significant socioeconomic and regional disparities in access to care and medication availability. Low income, multiple comorbidities, and residence outside Santiago were key predictors of moderate or severe access barriers, underscoring structural inequities within the health system.

### Comparison With Regional and Global Data

The observed prevalence of MDs in Cabo Verde (17 per 100,000) is notably lower than that reported in other African countries, such as Cameroon (3.2%) and Senegal (4.7%) [20, 21]. Similarly, the prevalence of PD in Cabo Verde (41 per 100,000) falls below rates reported in Nigeria (67) and Tunisia (436) and is significantly lower than the global rate estimated at 200 per 100,000 [6, 7]. While the lower prevalence in Cabo Verde may reflect true epidemiological differences, methodological factors, including the reliance on clinical records rather than population-based surveys, likely contributed to underestimation.

Temporal analysis revealed that while the age-standardized incidence of movement disorders in Cabo Verde has remained relatively stable over the last 3 years, the absolute number of diagnosed cases has increased. This divergence between crude and standardized rates likely reflects demographic aging and improved survival, rather than a genuine increase in underlying risk. Given the country’s expanding elderly population, now exceeding 7% of total inhabitants, these findings suggest that demographic and health system transitions are shaping the observed prevalence. A future longitudinal update including annual prevalence estimates will further clarify these dynamics.

As shown in Figure 3, the annual number of newly diagnosed cases increased gradually from the late 1980s onward, though the standardized incidence rate remained relatively stable. This pattern, occurring alongside urban growth and population aging, suggests that improvements in diagnostic capacity and health system accessibility likely contributed to the observed trends. Although Cabo Verde has experienced marked demographic and urban transitions over the past three decades, with urban residence rising to 77% and a rapidly aging population, our analysis of age-standardized incidence rates from 1986 to 2024 revealed a largely stable trend overall. This apparent stability, despite demographic growth and urbanization, likely reflects improvements in diagnosis and health service access rather than a true stagnation of new cases. Indeed, the observed upward trend in incidence among men may indicate greater occupational or environmental exposure, consistent with prior evidence linking pesticide and solvent exposure to movement disorders. The finding also suggests that increased awareness and diagnostic capacity in urban centers might have counterbalanced underrecognition in rural regions. Future studies incorporating longitudinal census data and detailed exposure histories will be crucial to disentangle demographic versus environmental contributions to MD incidence. Furthermore, the absence of specialized MD services and the concentration of neurology services in the capital region may result in substantial underdiagnosis, particularly in rural and remote areas.

### Health System Gaps and Barriers to Care

Beyond the epidemiological findings, the present study underscores critical health system challenges and barriers to care. Despite the majority of patients being on pharmacological treatment, nearly 70% reported moderate or severe difficulty accessing medications, primarily due to financial constraints, supply chain issues, and bureaucratic hurdles in the special import authorization process, which mirror challenges documented in other LMICs [1].

Additionally, the limited availability of multidisciplinary therapies, including physiotherapy, speech therapy, and psychological support, further compounds the challenges faced by MD patients. Although some services are available through private clinics, the high costs and geographic centralization on the island of Santiago place these services beyond the reach of many, especially those living in rural areas or on smaller islands. Notably, more than 60% of participants reported not receiving any form of therapy, and among those who did, many indicated that the number of sessions covered by insurance was insufficient. This aligns with evidence from other LMIC settings where rehabilitative services are fragmented, under-resourced, and rarely integrated into chronic care pathways for neurological conditions [1].

### Quality of Life Impacts and Lived Experiences

The study’s qualitative data provided valuable insights into the lived experiences of individuals with MDs in Cabo Verde. Participants highlighted the profound emotional, social, and functional impacts of these conditions, with over half reporting strong or extreme negative impacts on their lives. Commonly reported issues included motor difficulties, emotional distress (sadness, anxiety, irritability), sleep disturbances, and challenges with mobility and self-care. These findings are consistent with global research showing the multidimensional burden of MDs on patients’ physical health, mental wellbeing, social participation, and economic stability [22].

Moreover, the narratives of patients pointed to broader issues of stigma, marginalization, and invisibility within the health system and society at large. Many described feeling isolated, pitied, or misunderstood by healthcare providers, family members, and their communities. Such experiences highlight the need for public health interventions that go beyond clinical care to address the social determinants of health and promote social inclusion, patient empowerment, and community education.

### Policy Implications for LMICs

The results of this study offer important lessons for other LMICs facing similar epidemiological and health system challenges. First, they highlight the need for national health systems to recognize neurological disorders, including MDs, as priority areas within their NCD strategies.

Second, the study underscores the importance of strengthening data systems for neurological disorders in LMICs. We observed that health records in public and private institutions were often poorly maintained, leading to missing, lost, or duplicated clinical records, and inconsistencies between systems. Robust epidemiological data are essential for informing policy, planning services, and monitoring progress. Countries should invest in surveillance systems, population-based surveys, and health information systems that include MDs and other neurological conditions, ensuring data disaggregation by age, sex, socioeconomic status, and geographic location.

Third, the findings call for a paradigm shift toward integrated, people-centered models of care that address the complex needs of individuals with MDs across the continuum of care, from early detection and diagnosis to rehabilitation, psychosocial support, and palliative care. This includes training and equipping primary care providers to recognize and manage MDs, decentralizing services to district and community levels, and involving patients and caregivers in care planning and decision-making processes. In addition, strategies to improve access to affordable medications, such as inclusion in national essential medicines lists, pooled procurement, and subsidy programs, should be prioritized.

Finally, advocacy and public awareness campaigns are needed to reduce stigma, promote early help-seeking, and foster community-based support networks for people living with MDs. Multisectoral collaboration involving the health, social protection, disability, and civil society sectors will be key to addressing the multifaceted needs of this vulnerable population.

### Limitations

This study has several limitations. Although the at-risk population was derived from national census data rather than specific hospital catchment areas, the inclusion of all neurologists and primary referral institutions across both island regions supports the representativeness of the sample. Nonetheless, incomplete coverage of rural areas likely contributes to conservative estimates of national prevalence and incidence. Prevalence could not be calculated separately for 2022 and 2023 due to incomplete harmonization of clinical records from those years; however, the observed stability of age-standardized incidence coupled with rising case counts supports the same epidemiological interpretation.

The use of clinical records as the primary data source, while pragmatic in the context of resource constraints and absent registries, likely led to underestimation of true MD prevalence and incidence, particularly among populations with limited access to healthcare services. Although institutions spanned multiple islands and sectors, coverage was not exhaustive. Facilities without neurologists or with incomplete records were excluded, and patients seen only in community or private practice, especially by general practitioners, may not have been captured. As a result, the cohort likely overrepresents more complex or advanced cases, limiting the generalizability of some findings, particularly survey-based results. To strengthen external validity, future studies could compare coverage using a “positive control” neurological condition such as ALS, which is almost exclusively hospital-managed; regional comparisons of ALS incidence and prevalence could help estimate under-ascertainment in current hospital-based surveillance. Finally, although data were drawn from multiple islands and both public and private facilities, the participating institutions do not represent all healthcare settings nationwide, so the findings should be interpreted as indicative of national patterns rather than fully population-representative estimates. Additionally, while every effort was made to cross-reference data sources and minimize duplication, limitations in record-keeping systems and low participation in the online survey may have affected data completeness. Furthermore, the cross-sectional design precludes the establishment of causal relationships, and self-reported data on access and quality of life are subject to recall and response biases.

To mitigate these limitations, it’s important to note that data collection was based on clinical records of confirmed diagnoses, which ensured a minimum standard of diagnostic validity. Furthermore, all data sources were cross-referenced and harmonized to avoid duplication using multiple unique identifiers (identification number, date of birth, sex, and municipality of residence). Additionally, the questionnaire was designed with internal item redundancy to reduce the effects of recall and response bias, and regression analyses adjusted for relevant demographic and clinical covariates to minimize confounding While these measures do not eliminate all sources of under-ascertainment or bias, they strengthen the internal consistency of the dataset. Therefore, despite these limitations, the study represents the most comprehensive assessment of MDs in Cabo Verde to date and provides critical evidence to inform policy and programmatic responses in the country and similar LMIC contexts.

### Conclusions

This first country-level epidemiological study of MDs in Cabo Verde exposes critical public health and health systems gaps in addressing chronic neurological conditions in a small island LMIC. The findings highlight growing demographic vulnerability, profound access barriers, and inequities in care delivery. Although current prevalence remains lower than in many parts of Africa and globally, incidence rates are rising, especially among men. Despite relatively strong health infrastructure compared to other African nations, Cabo Verde’s services for MDs remain centralized, fragmented, and under-resourced, reflecting systemic weaknesses common to LMIC contexts. Policies should prioritize decentralizing neurological services, improving access to essential medications and therapies, and supporting multidisciplinary interventions to enhance the quality of life for individuals living with MDs.

Given the increasing burden of neurological disorders across sub-Saharan Africa and globally, Cabo Verde’s experience offers important lessons for other LMICs in designing equitable, sustainable, and integrated responses to address this growing public health challenge.
