# Supplementary material for: Movement Disorders in Cabo Verde: Epidemiology, Access Barriers, and Public Health Implications in an Aging Island Population
Source: Int J Public Health. 2026 Mar 3;71:1608728. doi: 10.3389/ijph.2026.1608728 (PMC13003321; doi:10.3389/ijph.2026.1608728)
Supplement: Supplementary file 1 [file DataSheet1.pdf]

* Required information

Research Questionnaire
Epidemiological Study of Movement Disorders in Cabo Verde

*
1. Date of Data Collection

_ _/_ _/_ _ _ _ [dd/mm/aaaa]

2. Identification of the interviewer/reviewer of medical files (Select one)

- ○ Cláudia Pires
- ○ Hedilaine Gomes
- ○ Leida Tolentino
- ○ Filipe Monteiro
- ○ Other - Specify __________

3. Study Participant's identification number

____

4. What is your sex? (Select one)

- ○ F
- ○ M

5. What is your current marital status? (Select one)

- ○ Single
- ○ Married
- ○ Separated
- ○ Divorced

Research Questionnaire
Epidemiological Study of Movement Disorders in Cabo Verde

- ○ Widowed
- ○ Cohabitating

6. Where do you currently live? (Select one option)

- ○ At home
- ○ Nursing Home
- ○ Extra-care housing
- ○ Hospital
- ○ Other-Specify __________

7. Where do you currently reside? (Select one option)

- ○ Urban Area (City)
- ○ Rural area (Countryside /Village)

7.1 Which island do you currently live in? (Select one option)

- ○ Santo Antão
- ○ São Vicente
- ○ São Nicolau
- ○ Sal
- ○ Boavista
- ○ Maio
- ○ Santiago
- ○ Fogo

Research Questionnaire
Epidemiological Study of Movement Disorders in Cabo Verde

- Brava

8. Do you currently have health insurance? (Select one option)

- Yes
- No
- Uncertain

NOTE : IF YOU ANSWER
Yes Go to Q8.1
No Go to Q9
Uncertain Go to page Q8.1

8.1 What is your health insurance? (Select one option)

- INPS
- Private (Employer)
- Private (Personal)
- Other-Specify __________

9. How many years in total did you study at a school, College or University (Select an option)

- No school
- Less than fourth grade
- Fourth grade to Sendo Year (Sixth grade)
- Incomplete Secondary School
- Complete Secondary School
- Incomplete University
- Bachelor’s Degree

Research Questionnaire
Epidemiological Study of Movement Disorders in Cabo Verde

- ○ Postgraduate degree (Master's, PhD ou more)

10. What best describes your main occupation? (Select one option)

- ○ Paid Work
- ○ Self-employed
- ○ Unpaid, volunteer or charity work
- ○ Student
- ○ Homemaker/Stay-at-home
- ○ Retired
- ○ Unemployed due to health reasons
- ○ Unemployed due to other reasons
- ○ Other -Specify __________

11. What is your current houseold income? (Select one option)

- ○ No income
- ○ Up to 10 000 CVE
- ○ 10-20 000 CVE
- ○ 20-35 000 CVE
- ○ 36-50 000 CVE
- ○ 51-75 000 CVE
- ○ 76-100 000 CVE

Research Questionnaire
Epidemiological Study of Movement Disorders in Cabo Verde

- ○ 101-150 000 CVE
- ○ 151-200 thousand Escudos
- ○ > 200 thousand Escudos
- ○ I'd rather not to answer

CLINICAL DATA

12. Are you being followed by a neurologist? (Select one option)

- ○ Yes
- ○ No

NOTE : IF YOU ANSWER
Yes Go to Q12.1
No Go to Q13

12.1 Who is the neurologist you currently see? (Select an option)

- ○ Dr. Antónia Rodrigues Fortes
- ○ Dr. Albertina Lima
- ○ Dr. João Xavier
- ○ Dr. Darius Lima
- ○ Dr. Ângela Fernandes
- ○ I don't remember
- ○ Other-Specify __________

13. Which Movement Disorder have you been diagnosed with?

* Required information

Research Questionnaire
Epidemiological Study of Movement Disorders in Cabo Verde

- ☐ Parkinson's Disease
- ☐ Dystonia
- ☐ Progressive Supranuclear Palsy (PSP)
- ☐ Huntington's Chorea
- ☐ Multisystemic Atrophy (MSA)
- ☐ Corticobasal Degeneration (CBD)
- ☐ Essential Tremor
- ☐ Don't Know
- ☐ Other-Specify ______________

14. When were you diagnosed with the aforementioned disorder? (Year of Diagnosis) (Select one option)

(Enter a value between 1930 and 2024)

---

15. When were you diagnosed with the aforementioned disorder? (month of Diagnosis) (Select one option)

- ◯ January
- ◯ February
- ◯ March
- ◯ April
- ◯ May
- ◯ June

Research Questionnaire
Epidemiological Study of Movement Disorders in Cabo Verde

- ○ July
- ○ August
- ○ September
- ○ October
- ○ November
- ○ December
- ○ I don't remember

16. How many first-degree relatives (parents, children, siblings), excluding yourself, have been diagnosed with movement disorder? (Select one option)

- ○ 0
- ○ 1
- ○ 2
- ○ 3
- ○ 4
- ○ 5+
- ○ I don't know

NOTE : IF YOU ANSWER **Q16**
0 Go to **Q17**
EVERYTHING ELSE Go to **Q16.1**

16.1. Which movement disorders have your first-degree relatives (parents, children, siblings), excluding yourself, have been diagnosed with?

- ☐ Parkinson's Disease
- ☐ Dystonia

Research Questionnaire
Epidemiological Study of Movement Disorders in Cabo Verde

- ☐ Progressive Supranuclear Palsy (PSP)
- ☐ Huntington's Chorea
- ☐ Multisystemic Atrophy (MSA)
- ☐ Corticobasal Degeneration (CBD)
- ☐ Essential Tremor
- ☐ Don't Know
- ☐ Other-Specify ______________

17. Do you have any other illness(es) or conditions(comorbidities).

- ☐ Hypertension (High Blood Pressure)
- ☐ Hyperlipidaemia (Elevated Lipids)
- ☐ Obesity
- ☐ Thyroid Disorder
- ☐ Arterial Fibrillation
- ☐ Cerebrovascular Disease
- ☐ Cerebral Vascular Accident (CVA)
- ☐ Diabetes w/ Sequelae
- ☐ Type 1 Diabetes
- ☐ Type 2 Diabetes
- ☐ Chronic Renal Failure
- ☐ Ulcers

- [ ] Congestive Heart Failure
- [ ] Rheumatism
- [ ] Peripheral Vascular Disease
- [ ] HIV/AIDS
- [ ] Any Malignant Neoplasm (Cancer)
- [ ] Metastatic Solid Tumour (Cancer)
- [ ] Tuberculosis
- [ ] Head Trauma
- [ ] Other- Specify ______________

17. What kind of neurological exams have you had? Select all that apply.

- [ ] Computerised Tomography (CAT)
- [ ] Magnetic Resonance Imaging
- [ ] Positron Emission Tomography (PET)
- [ ] None
- [ ] Other – Specify ______________

18- What kind of medications do you currently take?

- [ ] Antiparkinsonians
- [ ] Anticholinergics
- [ ] Neuroleptics
- [ ] Anticonvulsants

- ☐ Corticosteroids
- ☐ None
- ☐ Others ______________

NOTE: Answer the question below only if you selected (Q#18 **Antiparkinsonian**)

18.1 Antiparkinsonian Drugs

- ☐ Amantadine
- ☐ Levodopa-Benserazide
- ☐ Tolcapone
- ☐ Entacapone
- ☐ Selegelina
- ☐ Rasagiline
- ☐ Bromocriptine
- ☐ Pramipexole
- ☐ Pergolide
- ☐ Ropinirole
- ☐ Other-specify ______________

NOTE: Answer the question below only if you selected (Q#18 **Anticholinergics**)

- ☐ Biperiden
- ☐ Trihexyphenidyl
- ☐ Other-specify ______________

NOTE: Answer the question below only if you selected (Q#18 **Neuroleptics**)

Research Questionnaire
Epidemiological Study of Movement Disorders in Cabo Verde

18.3 Neuroleptics

- [ ] Risperidone
- [ ] Haloperidol
- [ ] Other-specify ______________

NOTE: Answer the question below only if you selected (Q#18 **Anticonvulsants**)

18.4 Anticonvulsants

- [ ] Phenobarbital
- [ ] Clonazepam
- [ ] Other-specify ______________

NOTE: Answer the question below only if you selected (Q#18 **Corticosteroids**)

18.5 Corticosteroids

- [ ] Fludrocortisone
- [ ] Other-specify ______________

NOTE: Answer the question below only if you selected (Q#18 **Other**)

18.6 Other

- [ ] Piracetam
- [ ] Baclofen
- [ ] Pizotifen
- [ ] Tetrabenazine
- [ ] Botulinum Toxin
- [ ] Penicillamine
- [ ] Trientine
- [ ] Other-specify______________

Research Questionnaire
Epidemiological Study of Movement Disorders in Cabo Verde

19. In general, how much difficulty do you (or your caretaker) have in accessing medications for your movement disorder? (Select one that applies)

- ○ None
- ○ Light
- ○ Moderate
- ○ Severe
- ○ Extreme or cannot
- ○ Not applicable

20. Do you currently have a co-payment from the National Social Security Institute (INPS) purchase of medication for your movement disorder? (select one that applies)

- ○ Yes
- ○ No
- ○ Uncertain

21. Do you currently follow any type of therapy (ies) (eg. Physiotherapy, Occupational therapy, etc.)? (Select one that applies)

- ○ Yes
- ○ No

NOTE: IF YOUR ANSWER **Q22** IS
Yes Go to **Q22.1**, **Q22.2** , **Q22.3**
No Go to **Q23**

22.1 What kind of therapy(ies) do you currently follow?

- ☐ Physiotherapy

Research Questionnaire
Epidemiological Study of Movement Disorders in Cabo Verde

- ☐ Phototherapy (Speech Therapy)
- ☐ Occupational therapy
- ☐ Psychology
- ☐ Nutrition
- ☐ Other-specify_____________

22.2 In general, how much difficult do you (or the person responsible for you) have in accessing the above-mentioned therapeutic services for your movement disorders? (select the option that applies)

- ○ None
- ○ Light
- ○ Moderate
- ○ Severe
- ○ Extreme or cannot
- ○ Not applicable

22.3 Do you currently have a co-payment from the National Social Security Institute (INPS) when going to therapy for your movement disorder? (select one that applies)

- ○ Yes
- ○ No
- ○ Uncertain

23. What could be the reasons for not following up on therapies? (Select all that apply)

- ☐ You do not need to

Research Questionnaire
Epidemiological Study of Movement Disorders in Cabo Verde

- ☐ Were not told about it
- ☐ Lack of financial resources
- ☐ Difficulties travelling
- ☐ Lack of therapy offers
- ☐ Other reasons-Specify ______________

LIFE SITUATION AND PROSPECTS

23. How many people live in your household? (Enter a number e.g 1,2,3, etc.)

Number of children	: ________
Number of siblings	: ________
Number of grandchildren	: ________
Father/Mother (1 or 2 parents)	: ________
Other(s)-Specify	: ________
I do not live at home (Insert '0')	: ________

24. Does anyone help look after you? Select all that apply.

- ☐ Children
- ☐ Siblings
- ☐ Grandchildren
- ☐ Father
- ☐ Mother

Research Questionnaire
Epidemiological Study of Movement Disorders in Cabo Verde

- ☐ Nurse
- ☐ Caretaker
- ☐ Other-Specify ______________

25. Do you or have you ever used Tobacco regularly? Tobacco consumed in any form, e.g. cigarettes, pipes, cigars, chewing tobacco and electronic cigarettes, including passively through a form of smoke. (Select one)

- ○ Yes
- ○ No
- ○ Cannot remember

NOTE: IF YOUR ANSWER **Q25** IS
Yes Go to **Q25.1**,
No Go to **Q26**
Cannot remember Go to **Q25.1**

25.1 How would you best identify yourself (Select one that applies)

- ○ Current smoker
- ○ Former Smoker (1-12 months ago)
- ○ Former Smoker (more than 12 months ago)
- ○ Passive Smoker
- ○ Former Passive Smoker (1-12 months ago
- ○ Former Passive Smoker (more than 12 months ago)

26. Do you have or have you ever consumed alcoholic beverages on a regular basis (grogue, beer, wine, punch liquor, whiskey, e.t.c) (Select one)

- ○ Yes
- ○ No

Research Questionnaire
Epidemiological Study of Movement Disorders in Cabo Verde

- ○ Cannot remember

NOTE: IF YOUR ANSWER **Q26** IS
Yes Go to **Q26.1**,
No Go to **Q27**
Cannot remember Go to **Q26.1**

26.1 IN THE LAST 12 MONTHS, how often did you drink 4 or more alcoholic drinks (women)/ 5 or more alcoholic drinks (men) in one day? (Select one that applies)

- ○ Daily or almost daily
- ○ Weekly
- ○ Monthly
- ○ Less than monthly

27. Do you use, or have you ever used any drugs, including cannabis (weed, 'padjinha'), cocaine or crack, heroin, methamphetamine, hallucinogens, ecstasy/MDMA, etc.? (Select one that applies)

- ○ Yes
- ○ No
- ○ Cannot remember

NOTE: IF YOUR ANSWER **Q27** IS
Yes Go to **Q27.1**,
No Go to **Q28**
Cannot remember Go to **Q27.1**

27.1 IN THE LAST 12 MONTHS, how often have you used any drugs, including cannabis, cocaine or crack, heroin, methamphetamine, hallucinogens, ecstasy/MDMA, etc.? (Select one that applies)

- ○ Daily or almost daily

Research Questionnaire
Epidemiological Study of Movement Disorders in Cabo Verde

- Weekly
- Monthly
- Less than monthly

28. Have you ever taken or are you currently taking any medication that was prescribed to improve your well-being, more than the prescribed amount or without a specific prescription for you? (Select one that applies)

- Yes
- No
- Cannot remember

NOTE: IF YOUR ANSWER **Q28** IS
Yes Go to **Q28.1**,
No Go to **Q29**
Cannot remember Go to **Q28.1**

28.1 IN THE LAST 12 MONTHS, how often have you abused a medicine (for example, have you used the medicine more than prescribed, or that it wasn't prescribed for you)? (Select one that applies)

- Daily or almost daily
- Weekly
- Monthly
- Less than monthly

29. Have you had direct exposure to chemicals used in agriculture (e.g. pesticides and herbicides), metals (iron, lead) or industrial chemicals such as solvents (e.g. trichloroethylene: TCE)? (Select one that applies)

- No

Research Questionnaire
Epidemiological Study of Movement Disorders in Cabo Verde

- ○ Yes
- ○ Uncertain
- ○ Less than a year
- ○ 1-2 years
- ○ 3-5 years
- ○ 6-10 years
- ○ More than 10 years

NOTE: IF YOUR ANSWER **Q29** IS
Yes Go to **Q29.1**,
EVERTHING ELSE GO TO **Q30**

29.1 Indicate the specific products to which you have been exposed (select all that apply):

- ☐ Rotenone (pesticide and insecticide)
- ☐ Paraquat (pesticide and insecticide
- ☐ Iron
- ☐ Copper
- ☐ Lead
- ☐ Manganese
- ☐ Polychlorinated biphenyls (PCBs; industrial chemical)
- ☐ Trichloroethylene (TCE; industrial chemical)
- ☐ Do not know
- ☐ Other- Specify _____________

30. Do you exercise? (Select one that applies)

Research Questionnaire
Epidemiological Study of Movement Disorders in Cabo Verde

- Yes
- No

NOTE: IF YOUR ANSWER **Q30** IS
Yes Go to **Q30.1** and **Q30.2**
No Go to **Q31**

30.1 How many minutes of physical activity do you do WEEKLY (e.g. walking, running, swimming, stationary bike, rowing machine, aerobic dance, gardening, cycling, etc.)? (Select one that applies)

- Uncertain
- 0-15 minutes
- 15-30 minutes
- 30-45 minutes
- 45-60 minutes
- 60-120 minutes (1-2 hours)
- 120-180 minutes (2-3 hours)
- 180+ minutes (more than 3 hours)

30.2 How often do you do physical activity each week? (Select one that applies)

- 1 x per week
- 2 x per week
- 3 x per week
- 4 x per week
- 5 x per week
- 6 x per week

Research Questionnaire
Epidemiological Study of Movement Disorders in Cabo Verde

- Everyday

31. IN THE LAST 30 DAYS, how often have you had problems sleeping, such as difficulty falling asleep, waking up frequently during the night, or waking up too early in the morning? (Select one that applies)

- Daily or almost daily
- Weekly
- Monthly
- Less than monthly
- Not Applicable

32. IN THE LAST 12 MONTHS, how much difficulty did you have smelling (olfaction)? (Select one that applies)

- None
- Light
- Moderate
- Severe
- Extreme or cannot
- Not applicable

COGNITION

33. IN THE LAST 30 DAYS, have you experienced any cognitive difficulties (processing information, e.g. difficulties with memory, concentration, and/or comprehension)? (Select one option that apply)

- Yes

Research Questionnaire
Epidemiological Study of Movement Disorders in Cabo Verde

- ◯ No
- ◯ Maybe
- ◯ Do not know

33.1 IN THE LAST 30 DAYS, how much difficulty did you have concentrating on something for at least 10 minutes? (Select one that applies)

- ◯ None
- ◯ Light
- ◯ Moderate
- ◯ Severe
- ◯ Extreme or cannot

33.2 IN THE LAST 30 DAYS, how much difficulty did you have learning a new task, e.g. learning to get to a new place? (Select one that applies)

- ◯ None
- ◯ Light
- ◯ Moderate
- ◯ Severe
- ◯ Extreme or cannot

MOBILITY

34. IN THE LAST 30 DAYS, have you experienced any motor difficulties (movements)? (Select one option that apply)

- ◯ Yes
- ◯ No

Research Questionnaire
Epidemiological Study of Movement Disorders in Cabo Verde

- ◯ Maybe
- ◯ Do not know

34.1 IN THE LAST 30 DAYS, how much difficulty did you have standing for long periods, such as 30 minutes? (Select one that applies)

- ◯ None
- ◯ Light
- ◯ Moderate
- ◯ Severe
- ◯ Extreme or cannot

34.2 N THE LAST 30 DAYS, how much difficulty did you have getting up and sitting down? (Select one that applies)

- ◯ None
- ◯ Light
- ◯ Moderate
- ◯ Severe
- ◯ Extreme or cannot

34.3 IN THE LAST 30 DAYS, how much difficulty did you have moving around your home? (Select one that applies)

- ◯ None
- ◯ Light
- ◯ Moderate
- ◯ Severe
- ◯ Extreme or cannot

* Required information

Research Questionnaire
Epidemiological Study of Movement Disorders in Cabo Verde

33.4 IN THE LAST 30 DAYS, how much difficulty did you have getting out of the house? (Select one that apply)

- ○ None
- ○ Light
- ○ Moderate
- ○ Severe
- ○ Extreme or cannot

33.5 IN THE LAST 30 DAYS, how much difficulty did you have walking a long distance, such as one kilometre [or equivalent]? (Select one option that apply)

- ○ None
- ○ Light
- ○ Moderate
- ○ Severe
- ○ Extreme or cannot

SELF-CARE

35. IN THE LAST 30 DAYS, did you find it difficult to look after yourself? (Select one that applies)

- ○ Yes
- ○ No
- ○ Maybe
- ○ Do not know

35.1 IN THE LAST 30 DAYS, how much difficulty have you had washing your whole body? (Select one that applies)

- ○ None

Research Questionnaire
Epidemiological Study of Movement Disorders in Cabo Verde

- Light
- Moderate
- Severe
- Extreme or cannot

35.2 IN THE LAST 30 DAYS, how much difficulty did you have getting dressed? (Select one that applies)

- None
- Light
- Moderate
- Severe
- Extreme or cannot

35.3 IN THE LAST 30 DAYS, how much difficulty did you have eating? (Select one that applies)

- None
- Light
- Moderate
- Severe
- Extreme or cannot

HUMOUR

36. IN THE LAST 30 DAYS, how often have you felt sad or unmotivated? (Select one option that applies)

- Daily or almost daily
- Weekly

Research Questionnaire
Epidemiological Study of Movement Disorders in Cabo Verde

- ○ Monthly
- ○ Less than monthly
- ○ Not Applicable

37. IN THE LAST 30 DAYS, how often have you felt irritable?
(Select one option that applies)

- ○ Daily or almost daily
- ○ Weekly
- ○ Monthly
- ○ Less than monthly
- ○ Not Applicable

38. IN THE LAST 30 DAYS, how often have you felt anxious?
(Select one that applies)

- ○ Daily or almost daily
- ○ Weekly
- ○ Monthly
- ○ Less than monthly
- ○ Not Applicable

DEALING WITH OTHERS

39. IN THE LAST 30 DAYS, have you experienced any difficulties in dealing with others?
(Select one that applies)

- ○ Yes
- ○ No
- ○ Maybe

- Do not know

39.1 IN THE LAST 30 DAYS, how much difficulty have you had dealing with people you don't know? (Select one that applies)

- None
- Light
- Moderate
- Severe
- Extreme or cannot

39.2 - IN THE LAST 30 DAYS, how difficult was it for you to get along with people close to you? (Select one that applies)

- None
- Light
- Moderate
- Severe
- Extreme or cannot

39.3 IN THE LAST 30 DAYS, how much difficulty have you had with sexual activities? (Select one that applies)

- None
- Light
- Moderate
- Severe

Research Questionnaire
Epidemiological Study of Movement Disorders in Cabo Verde

- Extreme or cannot

DAY-TO-DAY ACTIVITIES

40. IN THE LAST 30 DAYS, have you had any difficulty carrying out day-to-day activities? (Select one that applies)

- Yes
- No
- Maybe
- Do not know

40.1 IN THE LAST 30 DAYS, how much difficulty did you have carrying out your daily work? (Select one that applies)

- None
- Light
- Moderate
- Severe
- Extreme or cannot

PARTICIPATION

41. IN THE LAST 30 DAYS, did you experience any difficulties when taking part in activities (e.g. community or religious activities)? (Select one option that applies)

- Yes
- No
- Maybe
- Do not know

* Required information

Research Questionnaire
Epidemiological Study of Movement Disorders in Cabo Verde

41.1 IN THE LAST 30 DAYS, how much difficulty have you had participating in community activities (e.g. festivities, religious or other activities) in the same way that anyone else can? (Select one that applies)

- ◯ None
- ◯ Light
- ◯ Moderate
- ◯ Severe
- ◯ Extreme or cannot

41.2 N THE LAST 30 DAYS, how many problems have you had due to barriers or obstacles around you (physical or social)? (Select one that applies)

- ◯ None
- ◯ Light
- ◯ Moderate
- ◯ Severe
- ◯ Extreme or cannot

41.3 IN THE LAST 30 DAYS, how emotionally affected were you due to your health condition? (Select one that apply)

- ◯ Not affected
- ◯ Lightly affected
- ◯ Moderately affected
- ◯ Severely affected
- ◯ Extremely affected

41.4 IN THE LAST 30 DAYS, how much has your health affected your financial resources or those of your family? (Select one that applies)

- Not affected
- Lightly affected
- Moderately affected
- Severely affected
- Extremely affected

THE CONSEQUENCES OF DIFFICULTIES

42. IN THE LAST 30 DAYS, on how many days did these difficulties occur in general? (Select an option)

- 1
- 2
- 3
- 4
- 5
- 6
- 7
- 8
- 9
- 10
- 11
- 12
- 13
- 14

Research Questionnaire
Epidemiological Study of Movement Disorders in Cabo Verde

- ○ 15
- ○ 16
- ○ 17
- ○ 18
- ○ 19
- ○ 20
- ○ 21
- ○ 22
- ○ 23
- ○ 24
- ○ 25
- ○ 26
- ○ 27
- ○ 28
- ○ 29
- ○ 30

43. IN THE LAST 30 DAYS, for how many days were you totally unable to carry out your usual activities or work because of your health problem (motion sickness)? (Select one that applies)

- ○ 1
- ○ 2

* Required information

Research Questionnaire
Epidemiological Study of Movement Disorders in Cabo Verde

- ◯ 3
- ◯ 4
- ◯ 5
- ◯ 6
- ◯ 7
- ◯ 8
- ◯ 9
- ◯ 10
- ◯ 11
- ◯ 12
- ◯ 13
- ◯ 14
- ◯ 15
- ◯ 16
- ◯ 17
- ◯ 18
- ◯ 19
- ◯ 20
- ◯ 21
- ◯ 22
- ◯ 23

Research Questionnaire
Epidemiological Study of Movement Disorders in Cabo Verde

- 24
- 25
- 26
- 27
- 28
- 29
- 30

44. IN THE LAST 30 DAYS, for how many days did you cut down or reduce your usual activities or work because of your health problem (motion sickness), not counting the days you were totally incapacitated? (Select one that applies)

- 1
- 2
- 3
- 4
- 5
- 6
- 7
- 8
- 9
- 10
- 11
- 12

Research Questionnaire
Epidemiological Study of Movement Disorders in Cabo Verde

- ○ 13
- ○ 14
- ○ 15
- ○ 16
- ○ 17
- ○ 18
- ○ 19
- ○ 20
- ○ 21
- ○ 22
- ○ 23
- ○ 24
- ○ 25
- ○ 26
- ○ 27
- ○ 28
- ○ 29
- ○ 30

45. Finally, in your own words, how would you describe the main challenges you face in maintaining your quality of life in relation to your movement disorder?

___

___

* Required information

Research Questionnaire
Epidemiological Study of Movement Disorders in Cabo Verde
